# Supplementary material for: Who Presents Where? A Population-Based Analysis of Socio-Demographic Inequalities in Head and Neck Cancer Patients’ Referral Routes
Source: Int J Environ Res Public Health. 2022 Dec 13;19(24):16723. doi: 10.3390/ijerph192416723 (PMC9779534; doi:10.3390/ijerph192416723)
Supplement: Supplementary file 1 [file ijerph-19-16723-s001.zip › ijerph-2036615-supplementary.pdf]

Supplementary Materials

Who Presents Where? A population-based analysis of the socio-demographic inequalities in head and neck cancer patients' referral routes

Jennifer Deane, Ruth Norris, James O'Hara, Jo Patterson and Linda Sharp

Supplementary Figure S1 Route to diagnosis categorisation by each analysis
Supplementary Table S1: Demographic and clinical characteristics of all individual HNC diagnosis routes during 2006-2014

Supplementary Figure S1 Route to diagnosis categorisation by each analysis

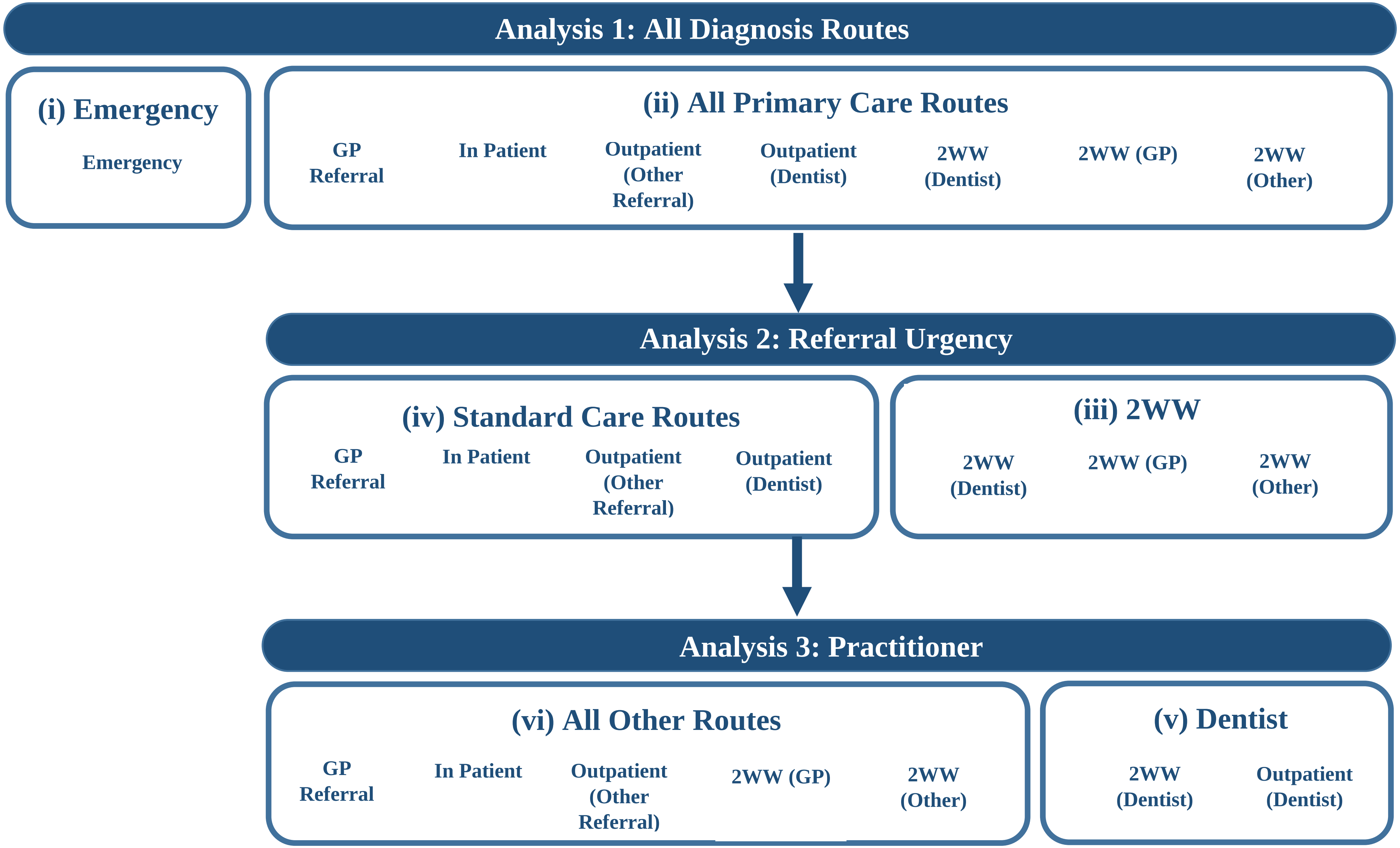

Supplementary Table S1. Demographic and clinical characteristics of all individual HNC diagnosis routes during 2006-2014.

Analysis 1: All Diagnosis Routes		Emergency	All Primary Care Routes						
Analysis 2: Referral Urgency			Standard Care Routes				2WW		
Analysis 3: Practitioner			All Other Routes			Dentist		All Other Routes	
	Overall (n=66,411; 100%)	Emergency (n=5,676; 8.5%)	GP Referral (n=21,803; 32.8%)	Inpatient (n=746; 1.1%)	Outpatient Other Referral (n=6,733; 10.1%)	Outpatient (Dentist) (n=5,305; 8.0%)	2WW (Dentist) (n=1,267; 1.9%)	2WW (GP) (n=22,741; 34.2%)	2WW (Other) (n=2,140; 3.2%)
**Age at Diagnosis**									
20-54 years	15,259 (23.0)	1,061 (18.7)	5,288 (24.3)	178 (23.9)	1,543 (22.9)	1,206 (22.7)	270 (21.3)	5,234 (23.0)	479 (22.4)
55-64 years	19,459 (29.3)	1,383 (24.4)	6,137 (28.1)	237 (31.8)	1,847 (27.4)	1,440 (27.1)	344 (27.1)	7,378 (32.4)	693 (32.4)
65-79 years	23,092 (34.8)	2,006 (35.3)	7,586 (34.8)	243 (32.6)	2,460 (36.5)	1,909 (36.0)	439 (34.6)	7,711 (33.9)	738 (34.5)
80+ years	8,601 (13.0)	1,226 (21.6)	2,792 (12.8)	88 (11.8)	883 (13.1)	750 (14.1)	214 (16.9)	2,418 (10.6)	230 (10.7)
**Sex**									
Male	46,241 (69.6)	4,028 (71.0)	15,050 (69.0)	526 (70.5)	4,568 (67.8)	2,863 (54.0)	773 (61.0)	16,860 (74.1)	1,573 (73.5)
Female	20,170 (30.4)	1,648 (29.0)	6,753 (31.0)	220 (29.5)	2,165 (32.2)	2,442 (46.0)	494 (39.0)	5,881 (25.9)	567 (26.5)
**Cancer Site**									
Oral Cavity[1]	22,620 (34.1)	1,316 (23.2)	5,831 (26.7)	204 (27.3)	2,284 (33.9)	4,523 (85.3)	1,106 (87.3)	6,802 (29.9)	554 (25.9)
Oropharynx	15,128 (22.8)	1,024 (18.0)	4,613 (21.2)	177 (23.7)	1,310 (19.5)	351 (6.6)	105 (8.3)	6,917 (30.4)	631 (29.5)
Larynx	15,885 (23.9)	1,686 (29.7)	6,228 (28.6)	189 (25.3)	1,593 (23.7)	37 (0.7)	6 (0.5)	5,631 (24.8)	515 (24.1)
Other[2]	12,778 (19.2)	1,650 (29.1)	5,131 (23.5)	176 (23.6)	1,546 (23.0)	394 (7.4)	50 (3.9)	3,391 (14.9)	440 (20.6)
**Deprivation Category**									
IMD 1 (Least Deprived)	10,417 (15.7)	656 (11.6)	3,438 (15.8)	187 (25.1)	1,294 (19.2)	1,030 (19.4)	239 (18.9)	3,279 (14.4)	294 (13.7)
IMD 2	12,260 (18.4)	808 (14.2)	4,147 (19.0)	158 (21.2)	1,292 (19.2)	1,117 (21.1)	234 (18.5)	4,137 (18.2)	367 (17.1)
IMD 3	13,245 (19.9)	1,062 (18.7)	4,431 (20.3)	156 (20.9)	1,260 (18.7)	1,094 (20.6)	260 (20.5)	4,537 (20.0)	445 (20.8)
IMD 4	14,217 (21.4)	1,319 (23.2)	4,731 (21.7)	129 (17.3)	1,343 (19.9)	1,046 (19.7)	251 (19.8)	4,934 (21.7)	464 (21.7)
IMD 5 (Most Deprived)	16,272 (24.5)	1,831 (32.3)	5,056 (23.2)	116 (15.5)	1,544 (22.9)	1,018 (19.2)	283 (22.3)	5,854 (25.7)	570 (26.6)
**Period of Diagnosis**									
2006-2008	19,623 (29.5)	1,875 (33.0)	7,193 (33.0)	384 (51.5)	2,312 (34.3)	1,467 (27.7)	240 (18.9)	5,588 (24.6)	564 (26.4)
2009-2011	22,206 (33.4)	1,850 (32.6)	7,452 (28.1)	175 (23.5)	2,399 (35.6)	1,836 (34.6)	366 (28.9)	7,447 (32.7)	681 (31.8)
2012-2014	24,582 (37.0)	1,951 (34.4)	7,158 (34.8)	187 (25.1)	2,022 (30.0)	2,002 (37.7)	661 (52.2)	9,706 (42.7)	895 (41.8)

Supplementary Table S1 Continued

Analysis 1: All Diagnosis Routes	Emergency	All Primary Care Routes						
**Analysis 2: Referral Urgency**		**Standard Care Routes**				**2WW**		
**Analysis 3: Practitioner**		**All Other Routes**			**Dentist**		**All Other Routes**	
**Overall** (n=66,411; 100%)	**Emergency** (n=5,676; 8.5%)	**GP Referral** (n=21,803; 32.8%)	**Inpatient** (n=746; 1.1%)	**Outpatient Other Referral** (n=6,733; 10.1%)	**Outpatient (Dentist)** (n=5,305; 8.0%)	**2WW (Dentist)** (n=1,267; 1.9%)	**2WW (GP)** (n=22,741; 34.2%)	**2WW (Other)** (n=2,140); 3.2%)
**Ethnicity**									
White	53.919 (81.2)	4,325 (76.0)	17,267 (79.2)	577 (77.3)	5,407 (80.3)	4,151 (78.2)	1,034 (81.6)	19,338 (85.0)	1,820 (85.0)
Non-White[3]	3,032 (4.6)	318 (5.6)	1,130 (5.2)	30 (4.0)	375 (5.6)	336 (6.3)	71 (5.6)	694 (3.1)	78 (3.6)
Unknown[4]	9,460 (14.2)	1,033 (18.2)	3,406 (15.6)	139 (18.6)	951 (14.1)	818 (15.4)	162 (12.8)	2,709 (11.9)	242 (11.3)
**Urban/Rural Category**									
Urban	54,704 (82.4)	4,889 (86.1)	17,913 (82.2)	593 (79.5)	5,569 (82.7)	4,262 (80.3)	1,023 (80.7)	18,628 (81.9)	1,827 (85.4)
Rural	11,707 (17.6)	787 (14.0)	3,890 (17.8)	153 (20.5)	1,164 (17.3)	1,043 (19.7)	244 (19.3)	4,113 (18.1)	313 (14.6)
**Stage**									
I	5,301 (8.0)	105 (1.8)	1,918 (8.8)	25 (3.4)	480 (7.1)	738 (13.9)	166 (13.1)	1,757 (7.7)	112 (5.2)
II	3,276 (4.9)	115 (2.0)	1,063 (4.9)	37 (5.0)	298 (4.4)	268 (5.1)	93 (7.3)	1,300 (5.7)	102 (4.8)
III	3,526 (5.3)	214 (3.8)	1,087 (5.0)	19 (2.5)	271 (4.0)	202 (3.8)	71 (5.6)	1,537 (6.8)	125 (5.8)
IV	13,043 (19.6)	1,235 (21.8)	3,312 (15.2)	96 (12.9)	1,008 (15.0)	925 (17.4)	350 (27.6)	5,600 (24.6)	517 (24.2)
Other[5]	41,265 (62.1)	4,007 (70.6)	14,423 (66.2)	569 (76.3)	4,676 (69.4)	3,172 (59.8)	587 (46.3)	12,547 (55.2)	1,284 (60.0)
**Grade**									
1 (Low)	5,589 (8.4)	323 (5.7)	1,926 (8.8)	49 (6.6)	568 (8.4)	802 (15.1)	136 (10.7)	1,649 (7.3)	136 (6.4)
2	25,074 (37.8)	1,770 (31.2)	7,728 (35.4)	265 (35.5)	2,175 (32.3)	2,382 (44.9)	663 (52.3)	9,281 (40.8)	810 (37.9)
3	17,851 (26.9)	1,424 (25.1)	5,551 (25.5)	207 (27.7)	1,708 (25.3)	854 (16.1)	289 (22.8)	7,101 (31.2)	717 (33.5)
4 (High)	603 (0.9)	66 (1.2)	237 (1.1)	8 (1.1)	69 (1.0)	14 (0.3)	5 (0.4)	175 (0.8)	29 (1.4)
Unknown[6]	17,294 (26.0)	2,093 (36.9)	6,361 (29.2)	217 (29.1)	2,213 (32.9)	1,253 (23.6)	174 (13.7)	4,535 (19.9)	448 (20.9)
**Comorbidities[7]**									
None	48,572 (73.1)	3,586 (63.2)	16,074 (73.7)	599 (80.3)	4,649 (69.0)	3,976 (74.9)	915 (72.2)	17,175 (75.5)	1,598 (74.7)
1	8,690 (13.1)	878 (15.5)	2,887 (13.2)	70 (9.4)	895 (13.3)	618 (11.6)	184 (14.5)	2,885 (12.7)	273 (12.8)
2+	9,149 (13.8)	1,212 (21.4)	2,842 (13.0)	77 (10.3)	1,189 (17.7)	711 (13.4)	168 (13.3)	2,681 (11.8)	269 (12.6)

[1]Includes palate; [2]Other cancer site refers to nasopharynx, hypopharynx, salivary glands, other sites and non-specific sites; [3]Non-White refers to other ethnic groups; [4]Unknown ethnicity refers to missing and unknown ethnicity; [5]Other stage refers to missing and unstageable tumours; [6]Unknown grade refers to unknown and missing tumour grades; [7]Measured using the Charlson Comorbidity Index; Abbreviations: GP: General practitioner; HNC: Head and neck cancer; IMD: Index of multiple deprivation; 2WW: Two week wait.
